# Modulatory role of macrophage migration inhibitory factor on cytokines and clinical features of sarcoidosis

**DOI:** 10.1038/s41598-022-21212-5

**Published:** 2022-10-07

**Authors:** Morvarid Elahi, Jaya Talreja, Brennen Steinbauer, Laura L. Koth, Lobelia Samavati

**Affiliations:** 1 Division of Pulmonary, Critical Care and Sleep Medicine, Department of Medicine, Detroit Medical Center, Wayne State University School of Medicine, 3 Hudson, 3990 John R Street, Detroit, MI 48201 USA; 2 Division of Pulmonary, Critical Care, Allergy and Sleep Medicine, Department of Medicine, University of California, San Francisco, San Francisco, CA 94143 USA; 3 Center for Molecular Medicine and Genetics, Wayne State University School of Medicine, Detroit, MI 48201 USA

**Keywords:** Inflammation, Immunology, Biomarkers, Medical research

## Abstract

Sarcoidosis is a systemic granulomatous disease of unknown etiology with significant heterogeneity in organ manifestations and clinical course. Subjects with sarcoidosis share several features such as, non-necrotizing granuloma, hypergammaglobulinemia, increased local and circulating inflammatory cytokines. Macrophage migration inhibitory factor (MIF) is a pluripotent chemokine modulating cellular function. Study included healthy controls (n = 28) and sarcoidosis patients (n = 65). Sera and BAL of sarcoidosis patients were collected and patients were followed longitudinally for 3 years, and demographics, stages, pulmonary function tests, and organ involvements were recorded. We evaluated MIF in the serum and bronchoalveolar lavage (BAL) fluid of sarcoidosis patients in association with clinical features and cytokines, IL-18, IL-10, IL-6, IFN-γ. We found serum MIF had a positive correlation with IL-10 and IFN-γ and % predicted total lung capacity (%TLC). Serum IL-18 had a significant positive correlation with serum lysozyme, but a negative correlation with %TLC and %DLCO. We identified two groups of sarcoidosis subjects with distinct clinical and cytokine features. A group with prominent extrapulmonary involvement, and low serum MIF, IL-10 and IFN-γ and a group with elevated serum MIF, IL-10 and IFN-γ levels. Our work provides understanding of phenotypic diversity in association with heterogeneity in cytokine landscape in sarcoidosis.

## Introduction

Macrophage migration inhibitory factor (MIF) is a pleiotropic cytokine produced by various cell types, including macrophages, neutrophils, lymphocytes, endothelial and epithelial cells. Additionally, MIF is released by the anterior pituitary and adrenal gland and functions as a hormone^1,2^. Initially, MIF was identified as lymphokines released by lymphocytes to inhibit the migration of macrophages and acquired its name owing to this function. MIF interacts with its membrane receptor, CD74, in association with CD44 to modulate macrophage function^3^. MIF acts as a proinflammatory cytokine but also has chaperon like functions and exhibits a thiol-protein oxidoreductase activity^4^. MIF acts also as a non-cognate ligand for the chemokine receptors, CXCR2, CXCR4, and CXCR7^2,5^. Therefore, MIF has a regulatory role for both innate and adaptive immune systems. MIF has a dichotomous action on immunity; MIF can act as a pro-inflammatory factor in response to stressors to potentiate of inflammatory signaling^6^. Others reported a protective role of MIF in the immunity against pathogens^1,7–9^. For instance, MIF-deficient mice exhibit higher burden of mycobacterial tuberculosis bacteria and succumb to earlier death^10^.

Sarcoidosis is a systemic granulomatosis disease of unknown etiology with variable clinical presentation and prognosis^11,12^. The granulomatous inflammation in sarcoidosis has been linked to enhanced T helper (Th1) function, B cells as well as macrophage activation^12,13^. Hypergammaglobulinemia is a frequent finding in sarcoidosis that may suggest active humoral immunity to unknown antigen(s). Studies indicate that B cells and increased IgG levels may play a role in the development of anergy or autoimmunity^14^. Because MIF acts in crosstalk between T and B lymphocytes, monocytes and macrophages^15^, investigating the role of MIF as a potential biomarker in sarcoidosis may prove to be beneficial.

In this work, we investigated the potential association of serum MIF and several important cytokines with sarcoidosis clinical features. Additionally, we compared levels of MIF and other cytokines in the lung compartment (BAL) and peripheral compartment (serum). Furthermore, we investigated if MIF levels were correlated with the levels of lysozyme, IL-10, IL-18, and interferon gamma (IFN-γ). We found that there were two distinct classes of sarcoidosis patients: (1) a group with high levels of MIF, and (2) a group with low MIF levels. The high MIF group was associated with elevated IL-10 and IFN-γ, whereas the low MIF group exhibited low IL-10 and IFN-γ. We found a significant positive correlation of serum lysozyme and IL-18 both of which were negatively associated with DLCO and FVC.

## Materials and methods

### Study design

The Committee for Investigations Involving Human Subjects at Wayne State University approved the protocol (IRB# 019111M1E) for obtaining alveolar macrophages by bronchoalveolar lavage (BAL) and blood by phlebotomy from control subjects and patients with sarcoidosis. All experiments were performed in accordance with relevant guidelines and regulations of investigation of human subjects. Sarcoidosis diagnosis was based on the ATS/ERS/WASOG statement^16^. The enrollment criteria were: (i) histologic demonstration of non-caseating granulomas with compatible clinical/radiographic features consistent with sarcoidosis, and (ii) exclusion of diseases capable of producing a similar clinical picture, such as fungus, mycobacteria. Exclusion criteria: (i) receiving immune suppressive medication (corticosteroid and/or other immune modulatory agents), (ii) had positive microbial culture in routine laboratory examinations or viral infection; or (iii) had known hepatitis or HIV infections or any immune suppressive condition. The criteria for enrollment in the control group were**:** (i) absence of chronic respiratory diseases (ii) absence of HIV or hepatitis infection. A total of 65 patients with sarcoidosis and 28 healthy controls participated in this study. The medical records of all patients were reviewed, and demographics, radiography stages, pulmonary function tests, and organ involvements were recorded as previously described^11^.

#### Pulmonary function test

PFTs were performed in patients following American Thoracic Society guidelines in a licensed laboratory^17^. All spirometry studies were completed using a calibrated pneumotachograph and lung volumes were measured in a whole-body plethysmograph (Jaeger Spirometry and Sensor Medics Vmax 22; VIASYS Respiratory Care, Inc., Yorba Linda, CA, USA) according to guideline^18^. The initial PFT’s were performed at the first attendance in the clinic. The PFTs completed in 3 years later were considered as the follow-up PFTs.

#### BAL collection

BAL was collected during bronchoscopy after administration of local anesthesia and before tissue biopsies, as previously described^11–13^. Briefly, a total of 200 mL of sterile saline solution was injected via fiberoptic bronchoscopy; the BAL fluid was retrieved, measured, and centrifuged. Samples were aliquoted and stored in -80º C freezer until use.

#### *Enzyme-linked immunosorbent assay (ELISA*)

The levels of MIF, IL-18, IL-10, IL-6 and IFN-γ lysozyme were measured by ELISA according to the manufacturer's instructions (ELISA Duo Kits; R&D Systems, Minneapolis, MN) as previously described^13^.

#### Measurement of immunoglobulin

Immunoglobulin measurement and subclassification performed in the diagnostic laboratory at Detroit Medical Center according to the standard procedure. Data were extracted from electronic medical records.

#### Statistical analyses

The statistical analyses were performed using the programming language R. For all of the box plots, a Shapiro–Wilk test was employed to see if a parametric or non-parametric test was appropriate. Furthermore, we evaluated the distribution and skewness of the data. The non-parametric Mann–Whitney U-test was used to determine the significance. A Spearman correlation test was applied to identify any association between the cytokines and mediators found to be altered in sarcoidosis patients, as compared with healthy controls. A *p* value of < 0.05 was considered statistically significant.

### Ethics approval and consent to participate

Written informed consent was obtained from all participants, and the study was approved by the Wayne State University, Institutional Review Board (IRB). All experiments were performed accordance to IRB. The IRB number of the study is 019111M1E.

## Results

### Serum MIF, IL-6, IL-18 and IFN-γ levels in sarcoidosis patients

We enrolled 93 subjects in this study comprising 65 sarcoidosis patients and 28 heathy subjects. Diagnosis of sarcoidosis was established based on American Thoracic Society criteria and exclusion of infection by examining samples for bacterial and viral infection^16^. All sarcoidosis subjects were ambulatory patients and sera were collected during first clinical encounters, when they were on no prior treatment. Clinical characteristics of the study subjects are demonstrated in Table 1.

**Table 1:** Subject demographics.

Characteristic	Patient subjects n = 65	Control subjects n = 28	*p*-value
Age, Years (mean ± SD)	48 ± 11	40 ± 10	0.47
Sex (female)	34 (61)	10 (66)	0.91
BMI, kg_._m^−2^ (mean ± SD)	30 ± 7	27 ± 5	0.38
**Race (n, %)**			
African–American	59 (90)	21 (75)	0.52
White	3 (4)	7(25)	0.31
Other	3 (4)	0	0.08
Smoker (PY)(mean ± SD)	5 ± 8	0	**0.00**
**Organ involvements (n, %)**			
Pulmonary Involvement	64 (98)	NA	–
Extra Pulmonary Involvement	48 (73)	NA	–
Skin	31 (47)	NA	–
Eye	24 (43)	NA	–
Heart	5 (9)	NA	–
CNS^a^	4 (7)	NA	–
Other	22 (33)	NA	–
**Initial chest radiograph stages (n, %)**			
Stage 0	2 (3)	NA	**–**
Stage 1	9 (16)	NA	**–**
Stage 2	31 (56)	NA	**–**
Stage 3	6 (10)	NA	**–**
Stage 4	4 (7)	NA	**–**
**Initial PFT**^b^**, mean (range**^c^**)**			
FVC^d^ (% predicted)	86 (44–123)	NA	**–**
FEV1^e^ (% predicted)	81 (27–121)	NA	**–**
FEV1/FVC (% predicted)	75 (36–98)	NA	**–**
TLC^f^ (% predicted)	80 (37–110)	NA	**–**
DLCO^g^ (% predicted)	65 (27–120)	NA	**–**

^a^Central nerve system, ^b^Pulmonary function test, ^c^Range, ^d^Forced vital capacity, ^e^Forced expiratory volume, ^f^Total lung capacity, ^g^Diffusing capacity of the lung for carbon monoxide.

Significant values are in bold.

**Figure 1:**
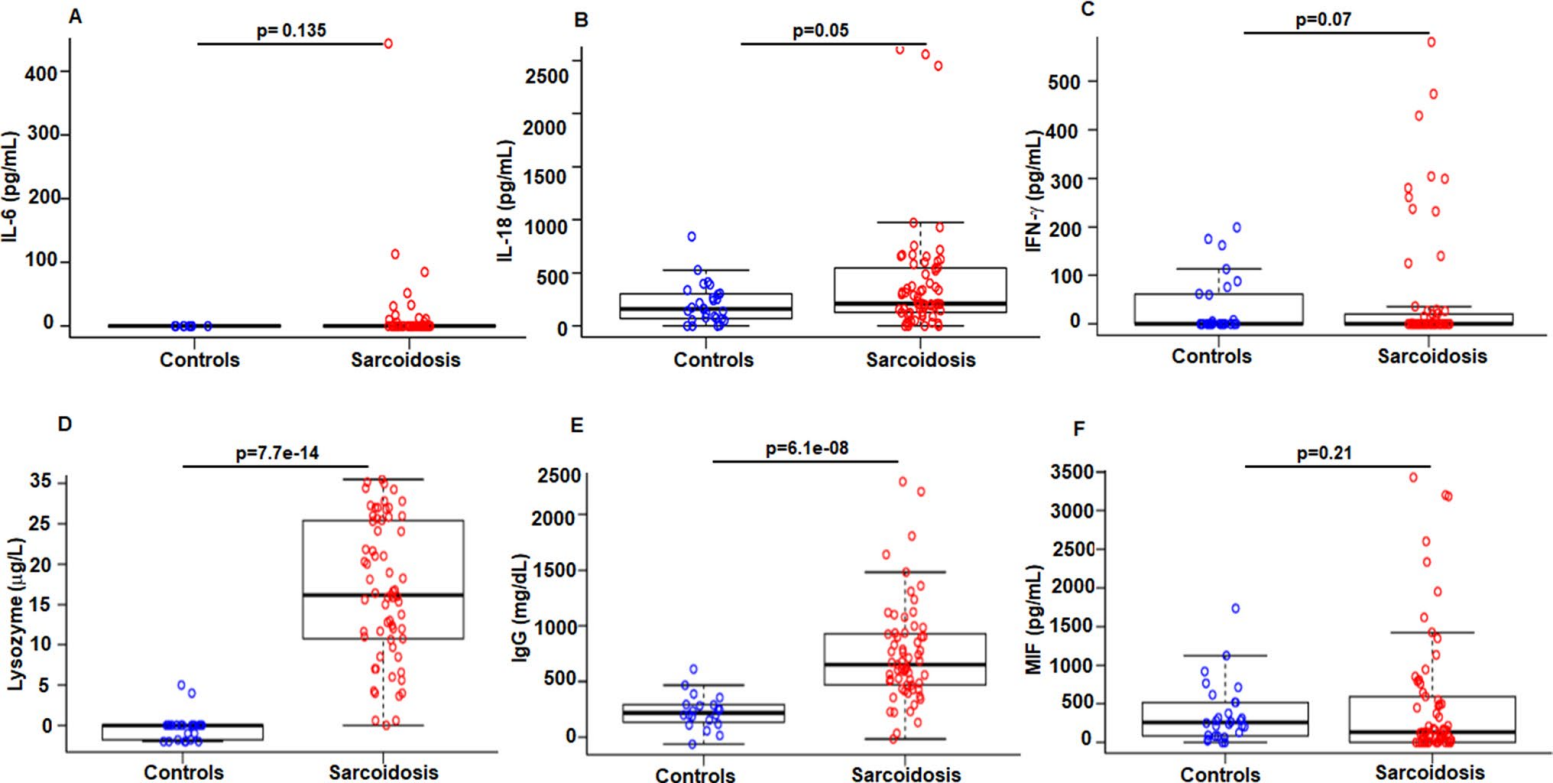
Comparison of levels of serum cytokines and biomarkers in sarcoidosis patients and healthy controls. The levels of cytokines and biomarkers were measured in serum samples via ELISA. The box plots were generated using programming language R and Mann–Whitney U test was applied. The central horizontal bar in the box plot represents the median value. There was no significant difference in levels of IL-6 between sarcoidosis patients and healthy controls (*p* = 0.135) (**A**). Sarcoidosis patients displayed significant higher levels of serum IL-18 (*p* = 0.05), (**B**). There was no significant difference in levels of IFN-γ (*p* = 0.07) (**C**). Sarcoidosis patients displayed significant higher levels of serum lysozyme (*p* = 7.7e−14) (**D**), and IgG (*p* = 6.1e−8) (**E**) as compared to healthy controls. (**F**) There was no significant difference in levels of MIF (*p* = 0.21), between sarcoidosis patients and healthy controls. *p* value < 0.05 was considered to be significant.

Production of cytokines is a well-regulated host defense mechanism against pathogens that is modulated by cellular cues and the cytokine milieu. To understand the regulation of group of cytokines implicated in sarcoidosis, we assessed the levels of IL-6, IL-18, IFN-γ and MIF in the serum samples of sarcoidosis patients (n = 65) and healthy controls (n = 28) via ELISA. IL-6 is a pro-inflammatory cytokine and has been suggested to play a role in the pathogenesis of sarcoidosis^19^. IL-18 belongs to IL-1 family of cytokines produced by monocytes and macrophages that has been shown to be increased in sarcoidosis patients^20,21^. Among 65 sarcoidosis subjects, only 11 patients show measurable IL-6 levels, and there was no significant difference (*p* = 0.135) in the levels of IL-6 between patients (median = 0) and healthy controls (median = 0) (Fig. 1A). In contrast, IL-18 was detectable in the sera from all participants (Fig. 1B). Sarcoidosis patients expressed significantly (*p* = 0.05) higher levels of IL-18 (median = 209 pg/mL) as compared to healthy controls (median = 160 pg/mL), despite two healthy controls exhibited higher IL-18 (842 and other 527 pg/mL). IFN-γ levels were higher (median = 0) in sarcoidosis as compared to the healthy group (median = 0) but did not reach statistical significance (*p* = 0.07) (Fig. 1C). There was a significant difference (*p* = 7.7e−14) in the lysozyme levels between controls (median = 5 µg/mL) and sarcoidosis subjects (median = 21.1 µg/L) (Fig. 1D). Serum IgG levels were significantly higher (*p* = 6.1e−08) in sarcoidosis patients (median = 1151 mg/dL) as compared to the healthy controls (median = 718 mg/dL) (Fig. 1E). We assessed MIF in the serum of our study subjects. Surprisingly, all healthy subjects had detectable MIF levels (median = 258 pg/mL). In fact, MIF was not measurable in a large number of patients or if it was detected, the levels were below the MIF mean values of healthy subjects, although some patients exhibited very high MIF levels. The median value of MIF levels in sarcoidosis patients were 130 pg/mL. The Box plot indicates a wide distribution of MIF values among sarcoidosis patients (Fig. 1F).

### Sarcoidosis patients with high MIF levels exhibit an increased IFN-γ and IL-10

**Figure 2:**
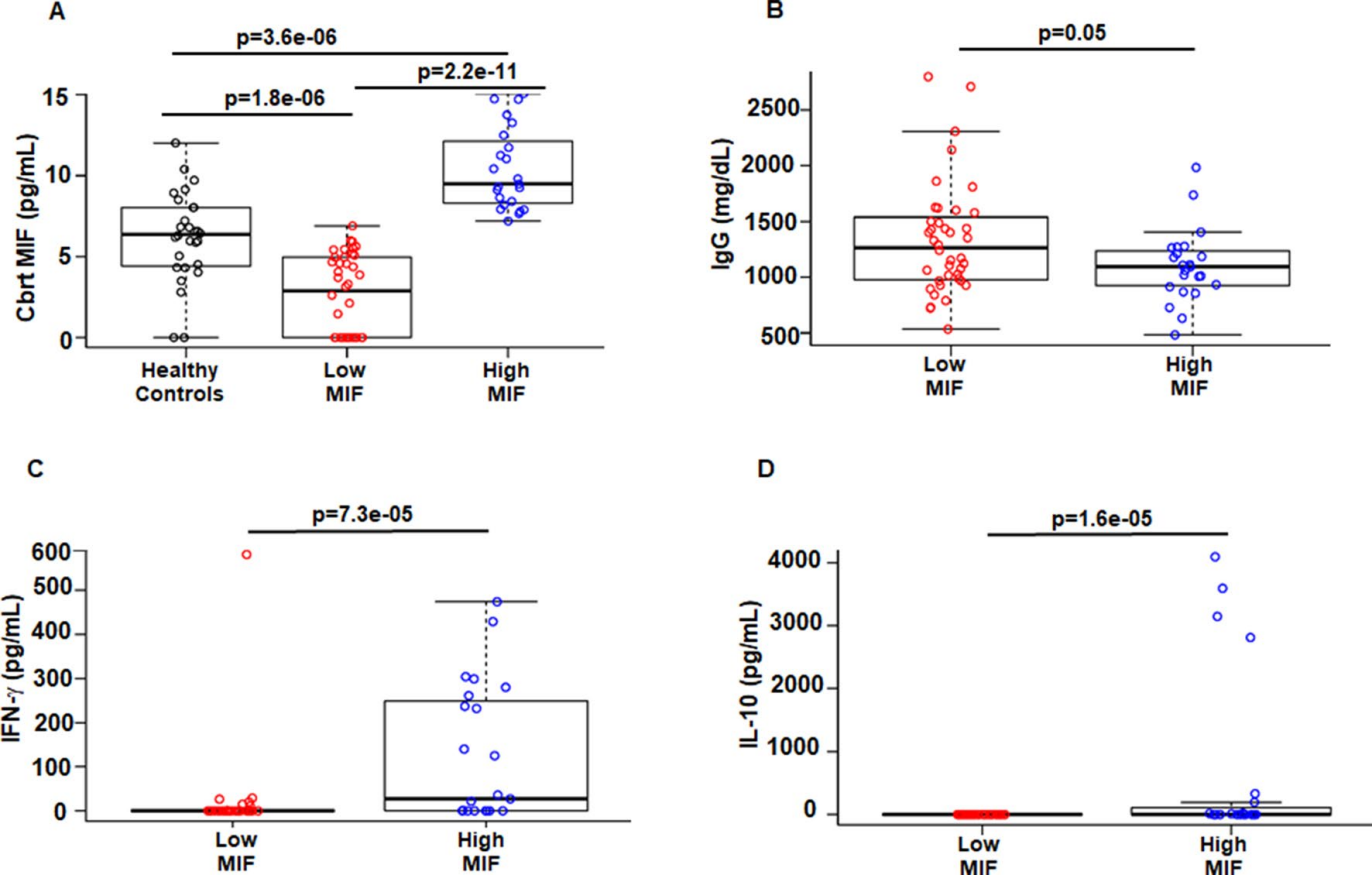
Classification of sarcoidosis patients based on serum MIF levels. Boxplots illustrate median values and interquartile range of each marker. Sarcoidosis patients with serum cbrt MIF values of less than 7.18 pg/mL were classified as low serum MIF subgroup, whereas MIF higher than 7.18 pg/mL were classified as high MIF subgroup. Healthy controls had significantly higher levels of MIF (*p* = 1.8e−06) as compared to low MIF subgroup, high MIF subgroup had significantly higher levels of MIF as compared to healthy controls (*p* = 3.6e−06) and low MIF subgroup (*p* = 2.2e−11) (**A**). Boxplot depicts the differences of median and interquartile range in serum IgG based on MIF subgrouping. Low serum MIF-group exhibited significant higher levels of serum IgG (*p* = 0.05) as compared to high MIF subgroup (**B**). Boxplot depicts the differences of median and interquartile range of serum IFN-γ. High serum MIF-group exhibited significantly higher levels of serum IFN-γ (*p* = 7.3e−05) (**C**). Boxplot depicts the differences of median and interquartile range of serum IL-10 between sarcoidosis MIF subgroups. Median values in low-MIF group were significantly (*p* = 1.6e−05) higher (**D**). *p* value < 0.05 was considered to be significant.

MIF has important immunomodulatory functions, both protective and detrimental roles, in the innate and adaptive immune system^2,8,15^. Because of wide distribution of MIF among sarcoidosis, we asked if there is a difference between subjects with elevated MIF versus subjects with unmeasurable or low MIF group in regards to cytokine values. The mean serum MIF-value in the healthy group was 370 pg/mL, we chose this value as the cut off value. Since the serum MIF values were not normally distributed, to reduce the skew and to reach a normal distribution, we transformed the values by cube root (cbrt) MIF. The cube root of the mean MIF was 7.18, based on which patients were classified in low and high serum MIF-groups and performed subgroup analysis. The box plot (Fig. 2A) shows the different group of subjects based on the serum (cbrt) MIF values corresponding to 370 pg/mL. The results in Fig. 2A shows that healthy controls (median = 6.3 pg/mL) have significantly higher levels of MIF (*p* = 1.8e−06) as compared to low MIF subgroup (median = 2.8 pg/mL). High MIF subgroup have significantly higher levels of MIF (median = 9.4 pg/mL) as compared to healthy controls (*p* = 3.6e−06) and low MIF subgroup (*p* = 2.2e−11). First, we analyzed the characteristics of these two groups in terms of clinical features and other clinical biomarkers. Clinical characteristics of patients stratified by low and high serum MIF levels are provided in Table 2. We found that the low serum MIF group was characterized by greater multiorgan involvements including, cardiac (*p* = 0.05) and CNS (*p* = 0.09) as compared to the high MIF group. Interestingly, patients with extensive skin disease exhibited elevated serum MIF levels (high MIF group). Hypergammaglobulinemia is a feature of sarcoidosis and elevated levels of IgG has been reported^22^. We evaluated if sarcoidosis cytokine profiles, CRP, and IgG are different in two MIF classification groups. Classification based on MIF values showed that patients with low MIF levels have elevated IgG levels (*p* = 0.05), (median = 1264 mg/dL) as compared to high MIF group (median = 1093 mg/dL) (Fig. 2B). IFN-γ is a pleotropic cytokine modulating both the innate and adaptive immune response against pathogens and plays a role in sarcoidosis^23^. IL-10 has potent anti-inflammatory activities by suppressing the granuloma formation^24^. We found that higher IFN-γ (*p* = 7.3e−05) and IL-10 (*p* = 1.6e−05) levels were almost exclusively observed in the high MIF group (Fig. 2C and D). The serum values for IFN-γ in low MIF group were median = 0 pg/mL; high MIF group were median = 27 pg/mL. The serum IL-10 values in low MIF group were median = 0 pg/mL; high MIF group were median = 0 pg/mL. In contrast, we found no significant differences in IL-18, lysozyme and CRP between low and high MIF subgroups (Table 2).

**Table 2:** Clinical characteristics based on serum MIF level.

Clinical characteristic	Low MIF serum n = 42(64%)	High MIF serum n = 23(35%)	*p*-value
Smoker (PY) (mean ± SD)	4 ± 8	2.5 ± 4	0.32
**Serum indicator (mean ± SD)**			
MIF^a^ (pg/mL)	65 ± 81	1324 ± 1058	**2.2e−11**
Interferon-γ (pg/mL)	16 ± 89	125 ± 155	**7.3e−05**
Interleukin-10 (pg/mL)	0	617 ± 1327	**1.6e−05**
Interleukin-18 (pg/mL)	350 ± 422	505 ± 687	0.90
Lysozyme (pg/mL)	21 ± 9	22 ± 7	0.84
CRP^b^ (mg/L)	15 ± 12	7 ± 6	0.12
IgG^c^ (mg/dL)	1335 ± 502	1100 ± 325	**0.05**
**Organ involvements (n, %)**			
Pulmonary Involvement	40 (95)	24 (95)	0.22
Extra Pulmonary Involvement	34 (81)	14 (52)	**0.05**
Skin	14 (33)	16 (68)	**0.03**
Eye	15 (46)	9 (39)	0.71
Heart	5 (15)	0	**0.05**
CNS^d^	4 (12)	0	0.09
Other	13 (37)	9 (34)	0.40
**Initial PFT**^**e**^** (mean, range**^**f**^**)**			
FVC^g^ (% predicted)	86 (44–123)	88 (44–118)	0.57
FEV1^h^ (% predicted)	81 (27–113)	81 (44–121)	0.91
FEV1/FVC (% predicted)	77 (36–98)	73 (43–95)	0.24
TLC^K^ (% predicted)	79 (54–110)	80 (37–107)	0.74
DLCO^s^ (% predicted)	65 (27–120)	65 (34–99)	0.91
**Follow up PFT (mean, range**^**f**^**)**			
FVC (% predicted)	86 (37–123)	92 (57–134)	0.27
FEV1 (% predicted)	80 (31–114	80 (44–135)	0.97
FEV1/FVC (% predicted)	74 (36–94)	70 (39–93)	0.25
TLC (% predicted)	76 (39–105)	82 (42–107)	0.11
DLCO (% predicted)	60 (22–112)	67 (49–103)	0.26

^a^Macrophage inhibitory factor, ^b^C-Reactive protein, ^c^Immunoglobulin G, ^d^Central nerve system, ^e^Pulmonary function test, ^f^Range, ^g^Forced vital capacity, ^h^Forced expiratory volume, ^K^Total lung capacity, ^s^Diffusing capacity of lung for carbon monoxide.

Significant values are in bold.

### Correlation of serum cytokines, lysozyme and IgG

**Figure 3:**
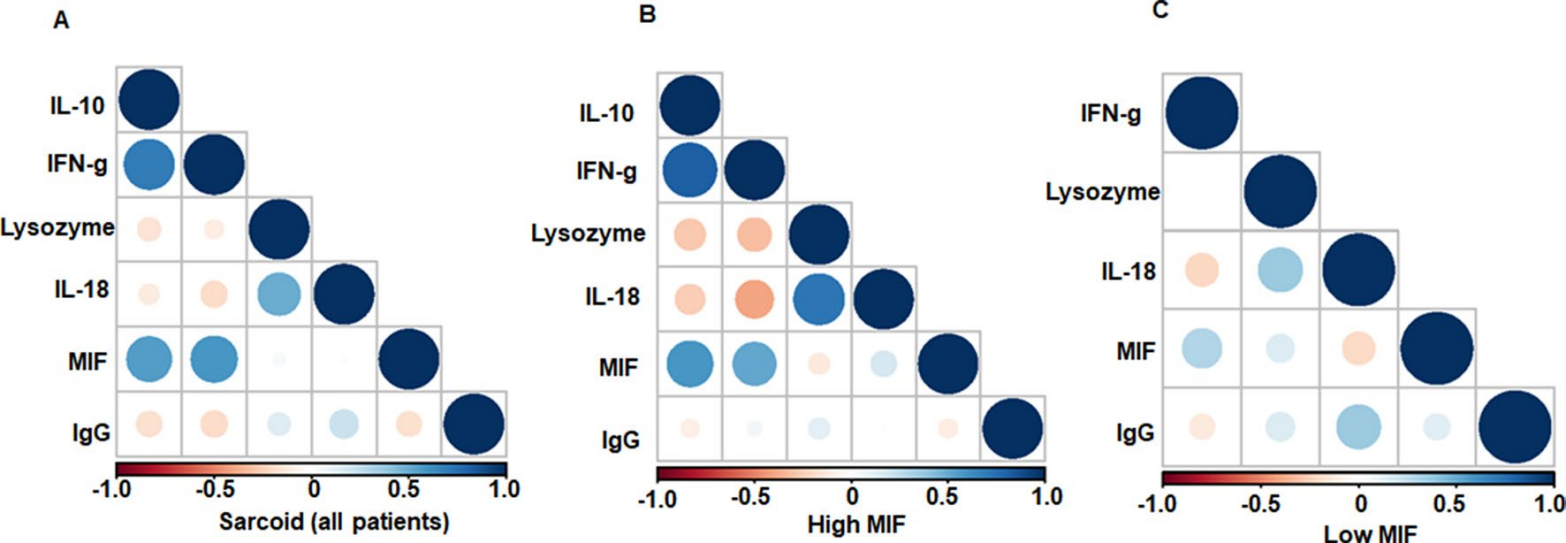
Correlation heatmap plots of serum cytokines and biomarkers in sarcoid patients. Correlation heatmaps of serum cytokines in sarcoid patients and low MIF and high MIF subgroup were made using statistical program R. The correlation and significance of mediators is depicted based on color (blue for positive correlation and pink for negative correlation) and size (larger depicts higher significance). Correlation heatmap of serum cytokines, IgG, and lysozyme among all sarcoidosis patients (**A**), high MIF subgroup (**B**) and low MIF subgroup (**C**).

Assessment of serum lysozyme is routinely used for evaluation of sarcoidosis^25,26^ and it is considered to be a marker for macrophage and monocytic activation^27^. A two-tailed non-parametric Spearman correlation was conducted to evaluate correlation among measured mediators including all patients regardless of MIF- subgrouping (Table 3). We found no significant correlation between serum MIF and IL-18 or lysozyme values. In contrast, there were a significant correlation between MIF and IFN-γ (r = 0.58; *p* < 0.001) (Table 3). We found that MIF values highly correlates with IL-10 values (r = 0.55; *p* value < 0.001). There was a significant positive correlation between IFN-γ and IL-10 (r = 0.68; *p* < 0.001) (Table 3). In contrast, we found a significant positive correlation between IL-18 and serum lysozyme levels (r = 0.49; *p* value < 0.001). Similarly, we determined the correlation between serum immunoglobulin G **(**IgG) and MIF and found that serum MIF negatively correlates (r = − 0.16) with IgG levels but did not reach statistical significance (*p* = 0.21) (Table 3). A correlation heatmap of serum cytokines, IgG, and lysozyme among all sarcoidosis subjects is demonstrated in Fig. 3A, depicting the correlation and significance of mediators based on color and size. Figure 3B and C show the correlation plots based on MIF subgroups. In contrast to the high MIF group, the low MIF group shows a lack of IL-10 production. Both groups displayed similar positive association between IL-18 and lysozyme. This association was more pronounced in the high MIF group (r = 0.72), whereas the association of IgG and IL-18 was more pronounced in low MIF group (r = 0.36) (Fig. 3B and C).

**Table 3:** Serum cytokines and biomarkers correlations.

	MIF	IFN-γ	IL-10	IL-18	Lysozyme	CRP	IgG
**MIF**							
Correlation	1	0.585**	0.547**	0.006	0.051	− 0.169	− 0.161
Sig		< 0.001	< 0.001	0.962	0.687	0.204	0.206
**IFN-γ**							
Correlation	0.585**	1	0.681**	− 0.151	− 0.082	− 0.06	− 0.185
Sig	< 0.001		< 0.001	0.23	0.518	0.654	0.146
**IL-10**							
Correlation	0.547**	0.681**	1	− 0.1	− 0.117	− 0.172	− 0.169
Sig	< 0.001	< 0.001		0.429	0.353	0.197	0.186
**IL-18**							
Correlation	0.006	− 0.151	− 0.1	1	0.490**	0.152	0.218
Sig	0.962	0.23	0.429		< 0.001	0.255	0.086
**Lysozyme**							
Correlation	0.051	− 0.082	− 0.117	0.490**	1	0.336**	0.131
Sig	0.687	0.518	0.353	< 0.001		0.01	0.307
**CRP**							
Correlation	− 0.169	− 0.06	− 0.172	0.152	0.336**	1	0.153
Sig	0.204	0.654	0.197	0.255	0.01		0.256
**IgG**							
Correlation	− 0.161	− 0.185	− 0.169	0.218	0.131	0.153	1
Sig	0.206	0.146	0.186	0.086	0.307	0.256	

*Correlation is significant at the 0.05 level (2-tailed).

**Correlation is significant at the 0.01 level (2-tailed).

### Correlation of serum MIF, IL-18, lysozyme and IgG with %TLC and %DLCO

**Figure 4:**
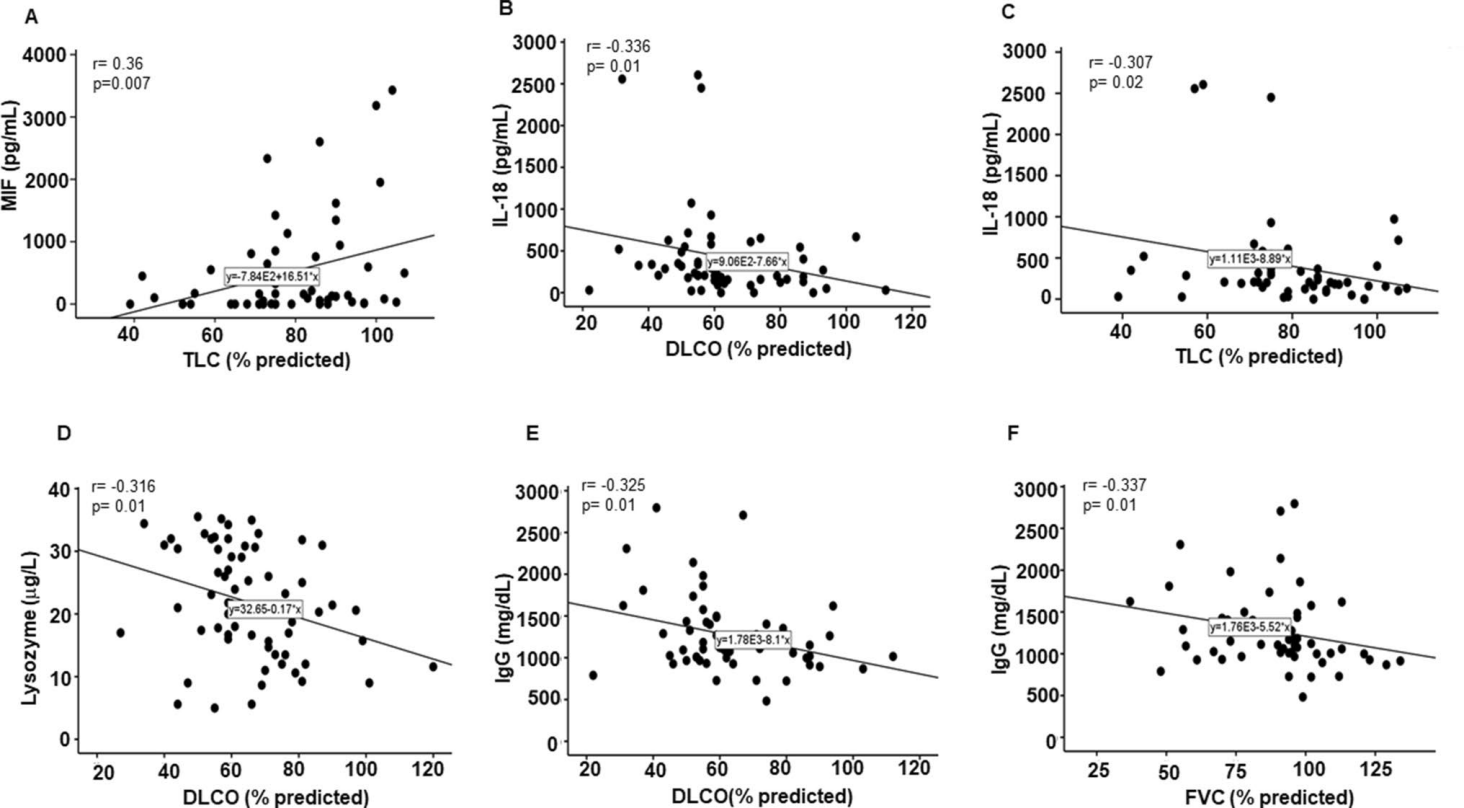
Correlation of serum cytokines and follow up PFT values in sarcoid patients. A correlation scatterplot depicts a positive association of MIF serum levels with %predicted TLC (r = 0.36, *p* = 0.007) (**A**). A correlation scatterplot of IL-18 showed a negative correlation with %DLCO (r = − 0.336, *p* = 0.01) (**B**);  and %TLC (r = − 0.307, *p* = 0.02) (**C**). A correlation scatterplot of lysozyme displayed a significant negative correlation with %DLCO (r = − 0.316, *p* = 0.01) (**D**). A correlation scatterplot of serum IgG levels displayed a significant negative correlation with %DLCO (r = − 0.325, *p* = 0.01) and (**F**) with %FVC (r = − 0.337, *p* = 0.01) (**E**). *p* value < 0.05 was considered to be significant.

Longitudinal measurements of lung function, such as % predicted total lung capacity (%TLC) and diffusion capacity of carbon monoxide (%DLCO) provide invaluable information about the progression of pulmonary sarcoidosis^28^. We evaluated the relationship of cytokine levels with PFT values: (1) PFTs at the time of sarcoidosis diagnosis and (2) PFTs obtained after 3 years follow-up. We found a negative and significant correlation between serum lysozyme and %DLCO and first PFT values (not shown). Next, we evaluated spearman correlation using %TLC values after 3 years follow up. Figure 4A shows that there is a positive, but significant association between MIF and %TLC (r = 0.36, *p* = 0.007). Scatter plots show a negative correlation of IL-18 with %DLCO (r = − 0.336, *p* = 0.01) as well as with %TLC (r = − 0.307, *p* = 0.02) (Fig. 4B and C). Scatter plot in Fig. 4D shows a negative correlation between lysozyme values and %DLCO (r = − 0.316, *p* = 0.01). Figure 4E and F shows that serum IgG exhibits negative correlation with %DLCO (r = − 0.325, *p* = 0.01) and with %FVC (r = − 0.337, *p* = 0.01). There was no correlation between CRP and PFT values.

### MIF, IL-18, IFN-γ, IL-6 and IL-10 levels in BAL of sarcoidosis patients

**Figure 5:**
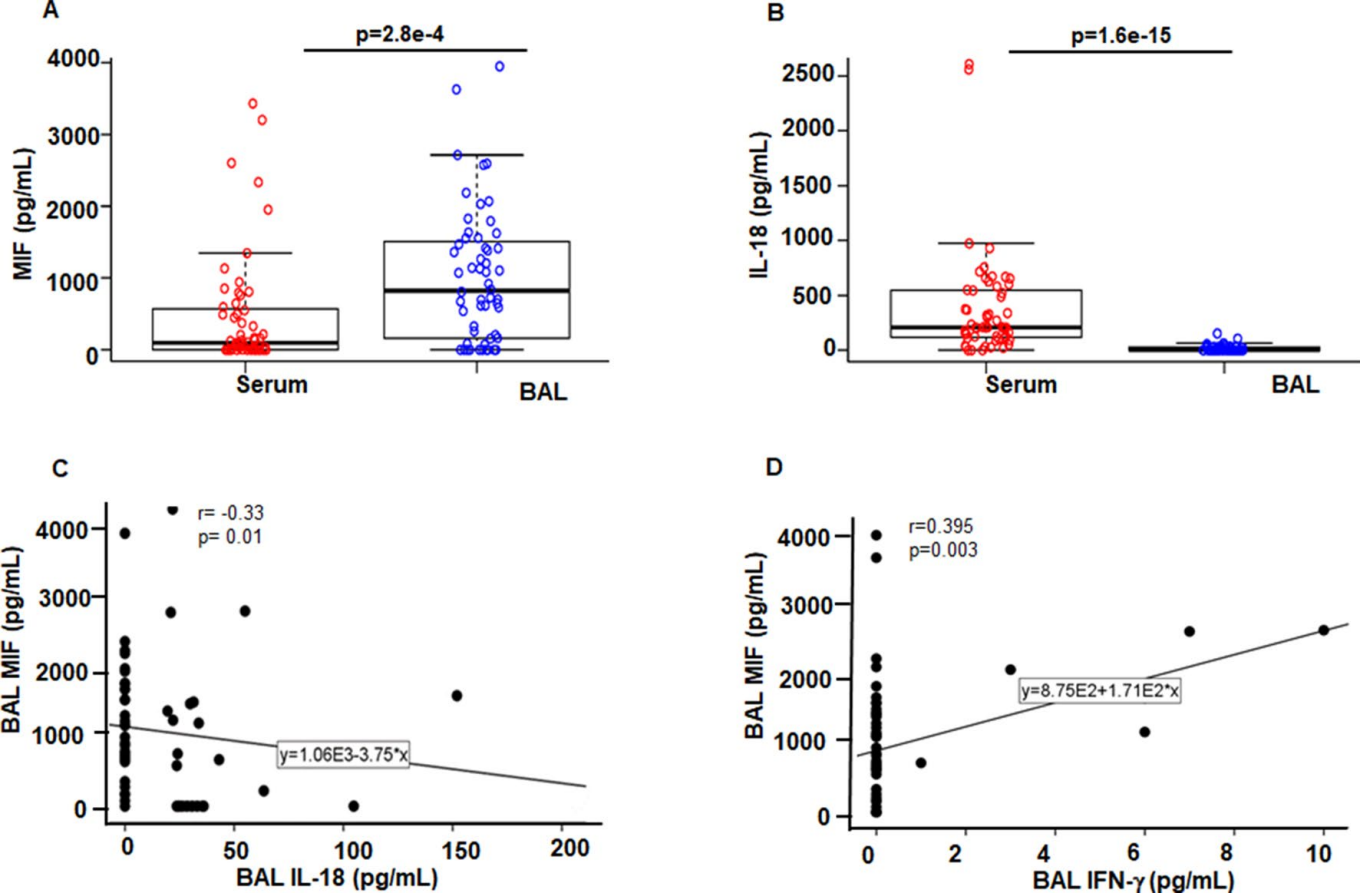
Comparison and correlation of MIF and IL-18 levels in serum and BAL of sarcoidosis patients. Boxplot depicts the differences of median and interquartile range of MIF. BAL samples displayed significant higher levels of MIF as compared to serum values using paired t-test (*p* = 2.8e−4) (**A**). Boxplot depicts the differences of median and interquartile range of IL-18 in serum and BAL samples. IL-18 level was significantly higher (*p* = 1.6e−15) in serum as compared to BAL (**B**). A correlation scatterplot of BAL IL-18 and BAL MIF values. There was a significant negative correlation between the levels of BAL IL-18 and BAL MIF (r = − 0.35, *p* = 0.02) (**C**). Correlation scatter plot displayed significant positive correlation between BAL MIF and BAL IFN-γ (r = − 0.395, *p* = 0.003) (**D**). p value < 0.05 was considered to be significant.

Sarcoidosis is a systemic disease with prominent lung involvement, but the degree of lung involvement and its progression varies among patients. It has been recognized that serum cytokine level may not mirror the cytokine values of the lungs or other tissues^29^. Next, we asked if these cytokines can be measured in BAL samples. All BAL and serum samples were collected, when the first diagnosis of sarcoidosis was established, and none were receiving any immune modulatory agents. The levels of MIF, IL-18, IFN-γ, IL-6 and IL-10 were measured in the BAL samples. About 80% BAL-samples had a detectable MIF with the mean value of 1007 ± 932 pg/mL. More than 50% of patients had a detectable IL-18 with the mean value of and 17 ± 21 pg/mL. IL-6 was detectable only in three BAL samples with the mean value of 4.4 pg/mL. The mean BAL IL-10 levels were 4 ± 8 pg/mL. Figure 5A shows the boxplot of MIF values in BALs and serum. The average of BAL MIF levels is significantly greater than the serum MIF level (*p* = 2.8e−4), serum MIF median = 98 pg/mL; BAL MIF median = 823 pg/mL (Fig. 5A). In contrast, the average of serum IL-18 levels was significantly greater than the BAL IL-18 values (*p* = 1.6e−15), serum IL-18 median = 207 pg/mL; BAL IL-18 median = 0 pg/mL (Fig. 5B). We found a negative correlation between MIF and IL-18 levels in BAL samples (r = − 0.35, *p* = 0.02) (Fig. 5C). In contrast, there was a positive correlation between BAL MIF and BAL IFN-γ (r = − 0.4, *p* = 0.003) (Fig. 5D). Table 4 shows the correlation of BAL cytokines (MIF, IFN-γ, IL-10 and IL-18).

**Table 4:** BAL cytokines correlations.

	MIF	IFN-γ	IL-10	IL-18
**MIF**				
Correlation	1	0.395**	0.239	− 0.336*
Sig		0.003	0.082	0.012
**IFN-γ**				
Correlation	0.395**	1	− 0.123	− 0.025
Sig	0.003		0.374	0.859
**IL-10**				
Correlation	0.239	− 0.123	1	− 0.096
Sig	0.082	0.374		0.49
**IL-18**				
Correlation	− 0.336*	− 0.025	− 0.096	1
Sig	0.012	0.859	0.49	

*Correlation is significant at the 0.05 level (2-tailed).

**Correlation is significant at the 0.01 level (2-tailed).

## Discussion

The immunopathogenesis of sarcoidosis remains poorly understood. Evidence suggests that exposure to unknown antigen(s) or environmental factors may lead to activation of macrophages, T and B cells^12,13,22,29^. Hypergammaglobulinemia is a frequent finding in sarcoidosis suggesting active humoral immunity^14,22^. Although some sarcoidosis clinical features, such as erythema nodosum, are associated with better prognosis^30^. It is not clear which cytokine pattern is associated with better clinical outcome. Therefore, cytokine immunophenotyping in association with clinical phenotypes may provide some insight in the cytokine characteristics and it could potentially improve therapeutic targeting.

Here, we found that there is a significant heterogeneity among sarcoidosis patients with respect to serum cytokine profile, including IL-18, IFN-γ, IL-10 and MIF. Serum IL-18 levels were measurable in almost all sarcoidosis patients. In contrast, IL-6 was measurable only in 18% of sarcoidosis patients. There were significant correlations between IL-18 and lysozyme levels (Table 3). Moreover, we found a significant negative correlation of IL-18 with % predicted TLC and DLCO at the time of diagnosis and in follow up PFT. We also measured cytokine levels in BALs obtained from the same patients at the time of diagnosis. In 2/3 of patients, MIF was measurable in the BAL samples. IL-18 was measurable in approximately half of the patients. We found a negative correlation between BAL MIF and IL-18, but a positive correlation with IFN-γ levels. Our data corroborates with previous data showing detectable IL-18 BAL levels of sarcoidosis subjects^20^.

MIF is a pleiotropic protein mediating its action through binding to receptor CD7 regulating innate and adoptive immunity^1,15,31^. The role of MIF in inflammatory disorders is diverse and it appears to be context dependent. Early investigations demonstrated that MIF plays a role in many forms of inflammatory diseases^15^. However, recent studies highlighted a protective role of MIF against oxidative stress-mediated DNA damage as well as various infection^1,7,8,10^. Based on recent experimental evidence, MIF may play a beneficial role by protecting from tissue senescence and damage in the lungs^32,33^. The role of MIF in sarcoidosis is poorly elucidated. We investigated if MIF levels can explain, at least in part, the phenotypic diversity in sarcoidosis patients. We identified a group of sarcoidosis patients with lower MIF levels, whereas another group of patients exhibited significantly higher serum MIF levels. Interestingly, subjects with low MIF levels tended to have more systemic and extra-pulmonary involvements and B symptoms such as weight loss, fatigue, night sweat, elevated IgG levels but lower IFN-γ levels and undetectable IL-10. Our data indicate that MIF levels correlate with improved follow-up % predicted TLC. Previous reports have indicated that MIF and activation of CD74 are important in reducing tissue injury and promoting tissue repair^1,32,34^. Increased IFN-γ have been reported in sarcoidosis patients^13,23^. IFN-γ could have protective and/or detrimental effects in the course of sarcoidosis^23^. IFN-γ orchestrates numerous protective functions in wound healing and repair^35^. IFN-γ has immunomodulatory effects through enhancing antigen processing and presentation, increasing leukocyte trafficking, boosting anti-microbial functions, and affecting cellular proliferation and apoptosis^35,36^. One previous study showed that elevated serum IFN-γ in sarcoidosis patients is associated with better lung function^23^. As MIF highly correlates with serum IFN-γ and IL-10 values, it is not clear whether the protective effects of MIF are direct or through IFN-γ and IL-10. It appears that IFN-γ and MIF can mutually regulate each other and synergistically modulate cell effector function^31,37^. Recent studies identified a critical role of MIF in pathogen clearance in Th1 mediated (mycobacterial tuberculosis)^10^ and Th2 mediated parasitic infection^31^. Interestingly, it has been shown that coactivation of IL-10 and IFN-γ downregulates the activation of antigen presenting cells and controls the pro-inflammatory responses via induction of regulatory T-cells (Treg)^38,39^. Another study found a defective immunosuppressive function of Treg in MIF knock out mice^40^. It has been reported a dysfunction of Treg in sarcoidosis^41^. It has been shown that MIF promotes AKT, ERK activation and alveolar repair by stimulating alveolar epithelial cell proliferation^33^. ERK activation is mechanistically linked to upregulation of IL-10 in dendritic cells and Treg^42^. It is possible that MIF enhanced ERK activation, promotes IL-10 production. Because previously, we have shown that activation of p38 MAPK but not ERK play a critical role in sarcoidosis inflammatory responses^11,12^. The MIF effect on ERK upregulation may in turn interferes with the activation of p38 MAPK. This notion is supported by Fallica, et.al. experimental data that MIF downregulates ASK mediated p38 activation in response to cigarette smoke^43^. As our current data in sarcoidosis indicate that MIF levels are positively associated with IFN-γ and IL-10, but negatively associated with IgG level. We speculate that elevated MIF improves antigen clearance and suppresses B cell function. Despite several studies in various respiratory diseases, including COPD and IPF or age related lung diseases^32,44^, it is not clear if MIF is detrimental or protective^2,7,32^. Therefore, both inhibition and activation of MIF have been considered as a therapeutic approach. Our study indicate that MIF has as a potential modulatory role in cytokine network and not necessarily detrimental in sarcoidosis. Further experimental studies need to elucidate the MIF role in IL-10 and IFN-γ as well as B cell function in sarcoidosis.

IL-18 has been implicated in many autoinflammatory diseases, including macrophage activation syndrome, inflammatory bowel disease, sarcoidosis and others^45,46^. IL-18 is a member of IL1cytokine family, and similar to IL-1β is expressed as a pro-cytokine and it needs to be cleaved intracellularly by caspase-1 or other protease to a mature, biologically active form^46^.

IL-18 is usually bound to IL-18 binding protein to neutralize its proinflammatory effect^45^. Previous studies have shown elevated IL-18 in sarcoidosis BAL fluids, in serum and in response to microbial constituents^46,47^. Our data showed measurable IL-18 both in BAL and serum of sarcoidosis patients. IL-18 in BAL negatively correlated with MIF BAL levels but it correlated positively with IFNγ. Our study showed a significant correlation between serum lysozyme and IL-18, both of which had a negative association with PFT values including the % predicted DLCO and FVC. Elevated serum lysozyme in granulomatous diseases, including sarcoidosis and tuberculosis is well established and suggests activation of myeloid and macrophages^25^.

It is important to note that due to systemic nature of sarcoidosis and that patients may have different degree of lung involvements; the disease cannot be judged by only the PFT values. Further larger and longitudinal studies need to be conducted to further elucidate the association between PFT values and cytokine network in sarcoidosis.

Our findings suggest two different cytokine patterns, the elevation of MIF is associated with increased IFN-γ and IL-10. Another group with low MIF and low IFN-γ and IL-10 but elevated IgG. Another important finding of our study is that elevation of serum lysozyme is associated with elevated IL-18. Although the BAL MIF was negatively associated with IL-18 levels, this negative association was less pronounced in serum, partly due to the differences in low and high MIF group. This finding may suggest contribution of different cell types: myeloid and macrophages versus T and B cells. Here, we advance our knowledge by identifying MIF as a systemic modulator of cytokine spectrum and clinical phenotype in sarcoidosis. These findings may be important in targeted drug development.

## Data Availability

All data generated or analysed during this study are included in this published article. The datasets used and/or analysed during the current study available from the corresponding author on reasonable request.

## Abbreviations

MIF: Macrophage migration inhibitory factor
IL: Interleukin
IFN-γ: Interferon gamma
Ig: Immunoglobulin
ELISA: Enzyme-linked immunosorbent assay
BAL: Bronchoalveolar lavage
CRP: C-reactive protein
DLCO: Diffusion capacity of carbon monoxide
TLC: Total lung capacity
PFT: Pulmonary function test
ERK: Extracellular signal-regulated kinase
MAPK: Mitogen-activated protein kinase
